# Sex-Dimorphic Differential Expression Profiles in the Brain of the Adult Chinese Soft-Shelled Turtle, *Pelodiscus sinensis*

**DOI:** 10.3390/ani14233426

**Published:** 2024-11-27

**Authors:** Pan Liu, Yanchao Liu, Junxian Zhu, Chen Chen, Liqin Ji, Xiaoli Liu, Xiaoyou Hong, Chengqing Wei, Xinping Zhu, Qiaoqing Xu, Jiang Zhou, Wei Li

**Affiliations:** 1 College of Animal Science and Technology, Yangtze University, Jingzhou 434020, China; lxp001222@163.com (P.L.); xuqiaoqing@163.com (Q.X.); 2 Key Laboratory of Tropical & Subtropical Fishery Resource Application & Cultivation of Ministry of Agriculture and Rural Affairs, Pearl River Fisheries Research Institute, Chinese Academy of Fishery Sciences, Guangzhou 510380, China; liuyanchao204@163.com (Y.L.); zhujunxian_1994@163.com (J.Z.); chenchen@prfri.ac.cn (C.C.); jiliqin@prfri.ac.cn (L.J.); liuxl@prfri.ac.cn (X.L.); hxy@prfri.ac.cn (X.H.); wcq1970@163.com (C.W.); zhuxinping@prfri.ac.cn (X.Z.)

**Keywords:** *Pelodiscus sinensis*, RNA sequencing, sex-dimorphic growth, brain

## Abstract

The Chinese soft-shelled turtle (*Pelodiscus sinensis*) is an economically important species in aquaculture, and its growth pattern is characterized by significant sexual dimorphism. However, the underlying molecular mechanisms of this phenomenon have mostly been investigated in the gonadal tissues of *P. sinensis*, and there are no articles on sex differentiation from the brain of *P. sinensis*. Here, we analyzed transcriptomes of the brains of adult male and female *P. sinensis* using high-throughput Illumina sequencing technology, establishing a set of differential genes and differential transcription factors. The data showed that there were 908 genes with significant differences in expression, of which 357 genes were up-regulated and 551 genes were down-regulated. We annotated using Gene Ontology (GO) and the Kyoto Encyclopedia of Genes and Genomes (KEGG), and screened some genes and pathways related to growth. There were 282 growth-related differential genes and 181 sex-related differential genes. We screened the genes’ growth hormone receptor (*GHR*) and vascular endothelial growth factor A (*VEGFA*), which may be related to the growth of *P. sinensis*. The pathways related to the growth and development of *P. sinensis* are the growth hormone synthesis, secretion, and action pathway; the MAPK (mitogen-activated protein kinase) pathway; and the calcium signaling pathway. In addition, through gene set enrichment analysis (GSEA), we screened out two genes, LIM homeobox protein 1 (*LHX1*) and fibroblast growth factor 7 (*FGF7*), which are related to both growth and sex differentiation, and through protein interaction analysis of these genes, we screened out eight genes, including *LHX1*, *FGF7*, *GHR*, fibroblast growth factor 4 (*FGF4*), *EGFR*, *BMP3*, GLI family zinc finger 2 (*GLI2*), and neuronal differentiation 1 (*NEUROD1*), and verified the expression levels of these eight genes in the brain of the *P. sinensis* by real-time quantitative PCR (qRT-PCR), which supported the reliability and accuracy of our transcriptome analysis. Our study provides a solid foundation for analyzing the mechanisms of sexual-dimorphic growth of *P. sinensis* and even other turtles.

## Simple Summary

The growth pattern of the Chinese soft-shelled turtle (*Pelodiscus sinensis*) is remarkably sexually dimorphic. However, the molecular mechanism of this phenomenon has been studied mainly in the gonadal tissues of *P. sinensis*, and no article has been published on the study of sex differentiation in the brain of *P. sinensis*. Here, we performed a whole transcriptome analysis of the brains of adult female and male *P. sinensis*. We obtained a set of differentially expressed genes and transcription factors, which we annotated with GO and KEGG, and screened some genes and pathways related to growth. In addition, through GESA analysis, we screened out two genes, *LHX1* and *FGF7*, which are related to both growth and sex differentiation, and through protein interaction analysis of these genes, we screened out eight genes and verified the expression levels of these eight genes in the brain of *P. sinensis* by qRT-PCR, which supported the reliability and accuracy of our transcriptome analysis. This study provides a solid foundation for analyzing the growth mechanism of sexual dimorphism in *P. sinensis* and even other turtles.

## 1. Introduction

The Chinese soft-shelled turtle (*Pelodiscus sinensis*), the number one aquaculture turtle in China, has a high economic value. The production of commercial turtles has exceeded 300,000 tons for eleven consecutive years, and in 2023 its annual production reaches 470,000 tons [1,2]. Fascinatingly, *P. sinensis* presents significant sex dimorphism in female and male growth patterns, as males grow at a rate 1.5 times faster than females [3,4]. However, the molecular mechanisms of this sex-dimorphic growth pattern, which is highly linked with sex in *P. sinensis*, is not well clarified. Previous studies mainly focused on exploring sex-linked master genes in gonads [5,6], and then obtaining pseudo-sex populations through the gain or loss of gene functions for the purposes of all-male breeding [7,8], thus utilizing the advantages of male growth in farming this species on an industrial scale. Conversely, the investigation of sex-linked growth mechanisms is often neglected. Therefore, digging massive sex-dimorphic genes associated with growth can contribute to elucidating the molecular mechanisms of sex-dimorphic growth in *P. sinensis*.

The brain is a crucial organ that regulates the growth of the body via the brain–pituitary–GH/IGF1 axis [9]. Meanwhile, the brain also regulates sex differentiation through the brain–pituitary–gonad axis [10]. Consequently, the brain is the key node linking growth and sex. In tongue sole (*Cynoglossus semilaevis*), transcriptome analysis of male and female brains identified 33 differential genes associated with sex-dimorphic growth [11]. Transcriptome sequencing of the goose pituitary obtained 290 differential genes, of which those enriched in the insulin pathway could increase protein and fat accumulation [12]. Comparative transcriptome analysis of four sexually size-dimorphic fishes screened growth-related differentially expressed genes *GH* and *CD22* in all brains, and *TNNT3B* and *MYPLF* in all muscles [13]. Nevertheless, there are few reports, to our knowledge, on the sex-dimorphic expression profiles in the brain of *P. sinensis*.

In this study, we analyzed the transcriptome of the brain tissues of adult female and male *P. sinensis* using Illumina RNA sequencing (RNA-Seq) technology to obtain their differential expression profiles. The differential expression genes and the differential transcription factors were then functionally annotated using Gene Ontology (GO) and Kyoto Encyclopedia of Genes and Genomes (KEGG) analyses. Meanwhile, a gene set enrichment analysis (GSEA) of male and female brain transcriptional profiles was also performed to identify candidate differential genes involved in both growth- and sex-related pathways. Potential genes and transcription factors interacting with candidate differential genes were analyzed using a protein–protein interaction (PPI) network. Finally, eight genes were randomly selected to verify the accuracy of RNA-Seq using quantitative reverse transcription PCR (qRT-PCR). Altogether, these data are expected to provide a useful genetic foundation for further studies on sex-dimorphic growth in *P. sinensis*.

## 2. Materials and Methods

### 2.1. Ethical Declaration

All experimental procedures involving turtles in this experiment were approved by the Experimental Animal Care and Ethics Committee of the Pearl River Fisheries Research Institute, Chinese Academy of Fishery Sciences. Furthermore, all experimental procedures were performed in accordance with relevant guidelines and regulations.

### 2.2. Sample Collection

The turtles used in this study were collected from the Wealth Xing Industrial Co., Ltd. (Huizhou, China). *P. sinensis* shows significant sexually dimorphic growth at the adult stage [3,4]. The ovaries of juvenile *P. sinensis* develop to the ooplasmic stage, and the seminiferous tubes in the spermathecae are inconspicuous, with spermatogonia in the tubes. At the age of 1, the ovaries *P. sinensis* develop to the primary follicular stage, and the number of spermatogonia increases in the seminiferous tubes of the spermathecae, and at the age of 2, the oviducts of *P. sinensis* contain mature ova and the seminiferous ovaries contain mature spermatogonia [14]. Therefore, we chose healthy two-year-old *P. sinensis* females and males (*n* = 9 each), which were anesthetized with an intraperitoneal injection of 0.05% MS-222 (20 mg/kg, Sigma, St. Louis, MO, USA) and sacrificed. Subsequently, the whole brains were rapidly collected and rinsed with 1 × PBS in diethylpyrocarbonate (DEPC) water, then stored in liquid nitrogen for total RNA extraction.

### 2.3. RNA Extraction and Quality Test

Total RNA was isolated using TRIzol reagent (Ambion, Carlsbad, CA, USA). The consistency and purity were tested by NanoQ™ (Thermo Scientific, Madison, WI, USA), and the quality was evaluated using an Agilent 2100 bioanalyzer (Agilent Technologies, Santa Clara, CA, USA).

### 2.4. Library Construction and High-Throughput Sequencing

High-quality RNA was enriched by Oligo (dT) beads and fragmented into short fragments using fragmentation buffer, then reversed into cDNA using NEBNext Ultra RNA Library Prep Kit for Illumina (New England Biolabs, Ipswich, MA, USA). The double-stranded cDNA fragments were end repaired, adenylated, and ligated to adapters. The ligation reaction was purified with AMPure XP Beads (Beckman Coulter, Brea, CA, USA) for selection of the inserted fragments. Finally, polymerase chain reaction (PCR) amplification and secondary purification were performed. Six high-quality cDNA libraries were assessed by Agilent 2100 Bioanalyzer and sequenced using Illumina NovaseqTM 6000 platform (Illumina, San Diego, CA, USA).

### 2.5. Transcriptome Assembly and Differential Gene Analysis

After filtering the low-quality raw data using Fastp (version 0.18.0) [15], an index of the reference genome was built, and paired-end clean reads were mapped to the *P. sinensis* genome [16] using HISAT2 2.4 [17]. The mapped reads of each library were assembled by StringTie v1.3.1 [18], and a FPKM (fragment per kilobase of transcript per million mapped reads) value was calculated to quantify transcript expression abundance through RSEM software [19]. The differential expression analysis of transcripts was performed by DESeq2 [20], of which the transcripts with an absolute fold change ≥1.5 and *p* value below 0.05 were considered differentially expressed genes (DEGs).

### 2.6. Functional Enrichment and GSEA Analysis

Gene Ontology (GO) [21] and the Kyoto Encyclopedia of Genes and Genomes (KEGG) database [22] were used for functional enrichment analysis of DEGs. The GO terms and KEGG pathways with Q values less than 0.05 were considered to be significantly enriched. In addition, we performed a gene set enrichment analysis (GSEA) using GSEA software (https://www.gsea-msigdb.org/gsea/index.jsp, accessed on 24 November 2024) [23] on all expressed genes from the female and male brains to identify whether a set of genes in specific GO terms showed significant differences between the two groups. Briefly, the gene expression matrix was inputted, and the genes were ranked according to the signal-to-noise normalization method [23]. The thresholds of significant enrichment were given as NOM *p* value < 0.05 and |NES| > 1 [24].

### 2.7. Protein–Protein Interaction Analysis

A protein–protein interaction (PPI) network was constructed with the default parameters using String v10 [25], which determined genes as nodes and interactions as lines in a network. The network was visualized using Cytoscape v 3.7.1 [26] to present a core gene biological interaction.

### 2.8. qRT-PCR Verification

In order to validate the accuracy of transcriptome sequencing, eight differentially expressed genes were randomly selected for qRT-PCR assay, including *LHX1*, *FGF7*, *GHR*, *FGF4*, *EGFR*, *BMP3*, *GLI2*, and *NEUROD1*. The qPCR volume was 20 μL, including 10 μL of iTaq Universal SYBR Green (BIO-RAD, Hercules, CA, USA), 1 μL of cDNA, 1 μL of each primer, and 7 μL of nuclease-free water. The qPCR cycling conditions for all target genes were as follows: 95 °C for 5 min; 40 cycles of 95 °C for 10 s, 60 °C for 20 s, and 72 °C for 20 s; and melting curve analysis at 95 °C for 15 s, 60 °C for 60 s, and 95 °C 15 s. Each sample was analyzed in triplicate. The *efα1* gene was used as an internal control [5], and gene expression levels were normalized using the 2^−ΔΔCt^ method [27].

## 3. Results

### 3.1. Sexual Dimorphism of Growth in P. sinensis

**Figure 1:**
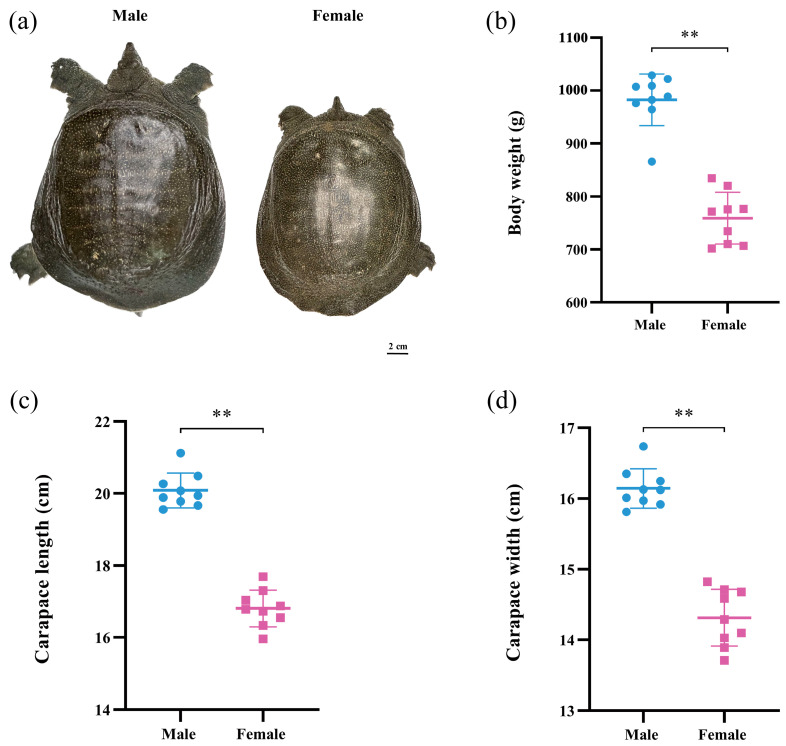
Morphological differences between male and female *P. sinensis*. (**a**) Comparison of body size in male and female individuals, (**b**) body weight (g), (**c**) carapace length (cm), (**d**) carapace width (cm) of nine males and nine females. Notation ** indicates significant differences between male and female individuals, *p* < 0.01.

In this study, significant differences in body weight and carapace size were observed between male and female *P. sinensis* (Figure 1). Figure 1a shows the distinct appearance of body size in adult male and female individuals, with males exhibiting a larger body size. In addition, the secondary sex characteristics of *P. sinensis* can also be observed, with the male turtle’s tail exceeding the skirt and the female’s tail inside the skirt. Figure 1b–d, respectively, display differences in body weight, carapace length, and carapace width between males and females. Measurements and statistical analyses revealed that the average body weight of male individuals (900–1100 g) was significantly higher than that of females (700–900 g) (*p* < 0.01). Additionally, the carapace length and width of male *P. sinensis* were significantly greater than those of females (*p* < 0.01). These results demonstrate the pronounced sexual dimorphism in *P. sinensis*, particularly in terms of body size and weight differences.

### 3.2. Screening for Differentially Expressed Genes

**Figure 2:**
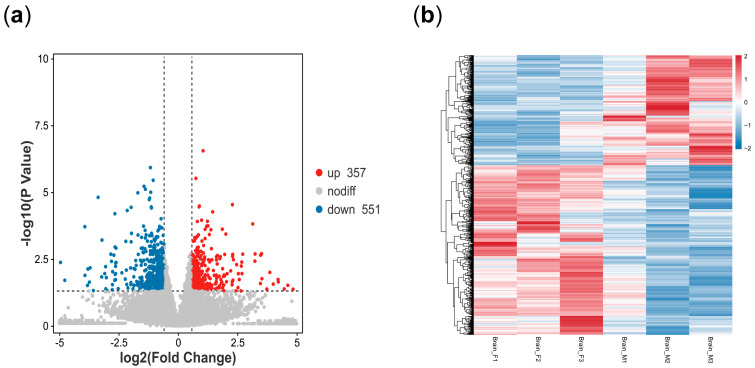
(**a**) Volcano map of differential expression genes in the brains of male and female *P. sinensis*. Up-regulated differential genes are labeled with red dots, down-regulated differential genes are labeled with blue dots, and non-differential genes are labeled with gray dots. (**b**) Clustered heatmap of differential expression genes in the brains of male and female *P. sinensis*. Rows and columns represent genes and samples. Color bars indicate gene expression levels from low (blue) to high (red).

This study obtained 5,597,172,355 to 6,405,607,451 clean data after removing the low-quality data with Q20 greater than 96.88%, Q30 greater than 91.65%, and GC content between 44.29 and 45.84%. Meanwhile, more than 88.50% of the clean data were aligned to the *P. sinensis* genome (Table 1). After normalization, there were 908 differentially expressed genes (DEGs) screened from the expression profiles of the female vs. male brains (Table S2), of which 357 were up-regulated and 551 down-regulated (Figure 2a). Hierarchical clustering analysis results showed that the expression patterns of DEGs were significantly different in female and male brains, indicating that a sex-dimorphic expression profile indeed exists in male and female brains of *P. sinensis* (Figure 2b). Among them, a number of genes expressed only in male brain tissue were also identified (Table S7), such as *ISL2*, *CNTNAP5*, and *Glyatl3*.

### 3.3. Functional Enrichment Analysis

**Figure 3:**
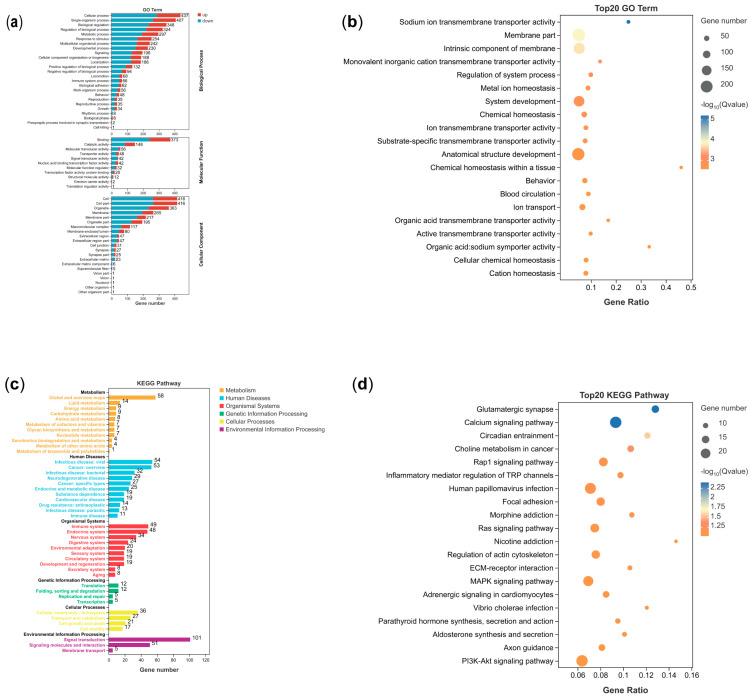
Analyses of functions and pathways of the differentially expressed genes in the brains of male and female *P. sinensis*. (**a**,**b**) represent the GO analysis of the differentially expressed genes and the top 20 enriched GO terms, respectively. (**c**,**d**) show the KEGG analysis of the differentially expressed genes and the top 20 enriched pathways, respectively.

GO and KEGG analyses were applied to annotate the biological functions and enriched pathways of the brains of male and female *P. sinensis*. GO enrichment analysis revealed that 908 DEGs were involved in 3619 GO terms (Table S3). GO terms were mainly classified into three categories, namely biological process, cellular component, and molecular function. All GO terms were mainly enriched in the cellular process of the biological process, the binding of the molecular function, and the cell of the cellular component (Figure 3a). Eighty-seven GO pathways were significantly enriched, including the sodium ion transmembrane transporter activity (GO:0015081), the membrane part (GO:0044425), and the intrinsic component of the membrane (GO:0031224). The sodium ion transmembrane transporter activity pathway had the highest significance among all top 20 GO-analyzed pathways (Figure 3b).

Meanwhile, KEGG terms were mainly classified into six categories, namely metabolism, human disease, organismal systems, genetic information processing, cellular processes, and environmental information processing. The KEGG enrichment results showed that all DEGs were contained to 283 KEGG pathways (Table S4) and all KEGG terms were mainly enriched in the global and overview maps of metabolism, infectious disease: viral of human disease, immune system of the organismal systems, translation of genetic information processing, cellular community eukaryotes of cellular processes, and signal transduction of environmental information processing (Figure 3c). Among them, the glutamatergic synapse (ko04724), the calcium signaling pathway (ko04020), the circadian entrainment (ko04713), and the choline metabolism in cancer (ko05231) were significantly enriched. Among all the top 20 KEGG-analyzed pathways, the glutamatergic synapse pathway had the highest significance and the calcium signaling pathway the second-highest significance. Moreover, the number of genes in the calcium signaling pathway was also higher (Figure 3d).

### 3.4. GSEA Analysis

**Figure 4:**
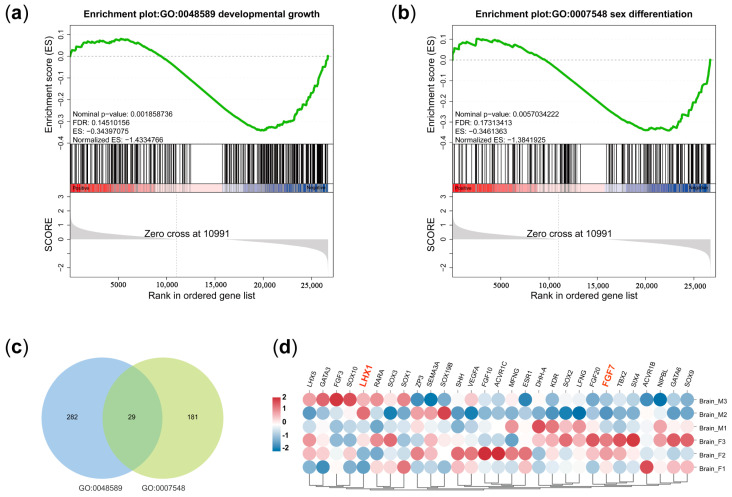
GSEA analysis identifies growth- and sex-related genes. (**a**,**b**) represent significantly enriched plot of developmental growth and sex differentiation, respectively. (**c**,**d**) show a Venn diagram and a cluster heatmap of genes that are commonly involved in growth- and sex-related GO terms, respectively.

To further explore the candidate genes that function in growth and sex, we performed a GSEA analysis for all expressed genes. The results show that a total of 837 GO terms were markedly enriched (Table S5), among which the growth-related pathway of developmental growth (GO:0048589, Figure 4a) and the sex-linked pathway of sex differentiation (GO:0007548, Figure 4b) were significantly down-regulated in the female brains. Venn diagram analysis demonstrated that there were 29 genes involved in both growth and sex differentiation in *P. sinensis* brains (Figure 4c). More importantly, only two genes (LHX1 and FGF7) were differentially expressed in the female and male brains (Figure 4d and Table S2).

### 3.5. Characterization of Differentially Expressed Transcription Factors

**Figure 5:**
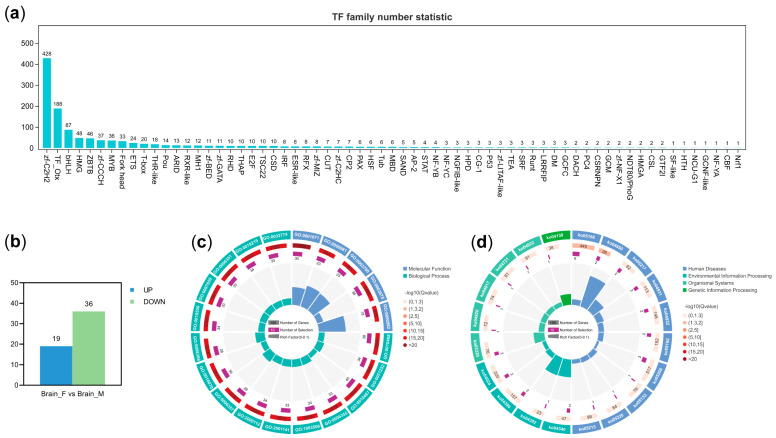
Identification and characterization of transcription factors. (**a**,**b**) represent bar charts of transcription factor family classification and differentially expressed transcription factors, respectively. (**c**,**d**) show GO analysis and KEGG analysis of differentially expressed transcription factors, respectively.

A total of 1244 transcription factors (TFs), belonging to 65 TF families, were identified in the gene set expressed in female and male brains (Table S6). The top three transcription factor families were zf-C2H2, TF_Otx, and bHLH (Figure 5a). Differential expression analysis showed that a total of 55 TFs were differentially expressed, of which 16 were up-regulated and 36 were down-regulated (Figure 5b). Moreover, all differentially expressed TFs were enriched in 1360 GO terms and 29 KEGG pathways. The top 3 significantly enriched GO terms were the nucleic acid binding transcription factor activity (GO:0001071), the RNA polymerase II transcription factor activity, sequence-specific DNA binding (GO:0000981), and transcription factor activity, sequence-specific DNA binding (GO:0003700) (Figure 5c). Among the KEGG pathways, the herpes simplex virus 1 infection (ko05168) and the maturity onset diabetes of the young (ko04950) were significantly enriched (Figure 5d).

### 3.6. Protein Interaction Network of LHX1 and FGF7

**Figure 6:**
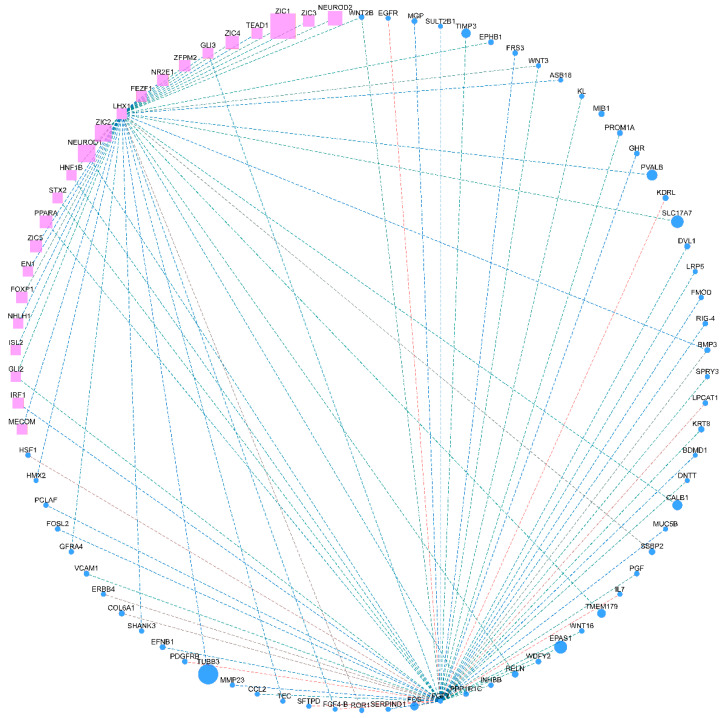
Protein interaction network of genes *LHX1* and *FGF7*. Pink squares and blue circles indicate interacting transcription factors and proteins, respectively. Connecting lines indicate potential interactions.

A PPI interaction network was constructed for further investigation of the interaction between two candidate genes, *LHX1* and *FGF7*, and all the differential genes (Figure 6). *LHX1* and *FGF7* interacted with 39 and 49 genes, respectively, which contained 23 transcription factors. Also, *LHX1* and *FGF7* co-acted with eight genes, including *WNT2B*, *WNT3*, *FGF4*, *GLI2*, *GLI3*, *HNF1B*, *NEUROD1*, and *BMP3*.

### 3.7. qPCR Verification

**Figure 7:**
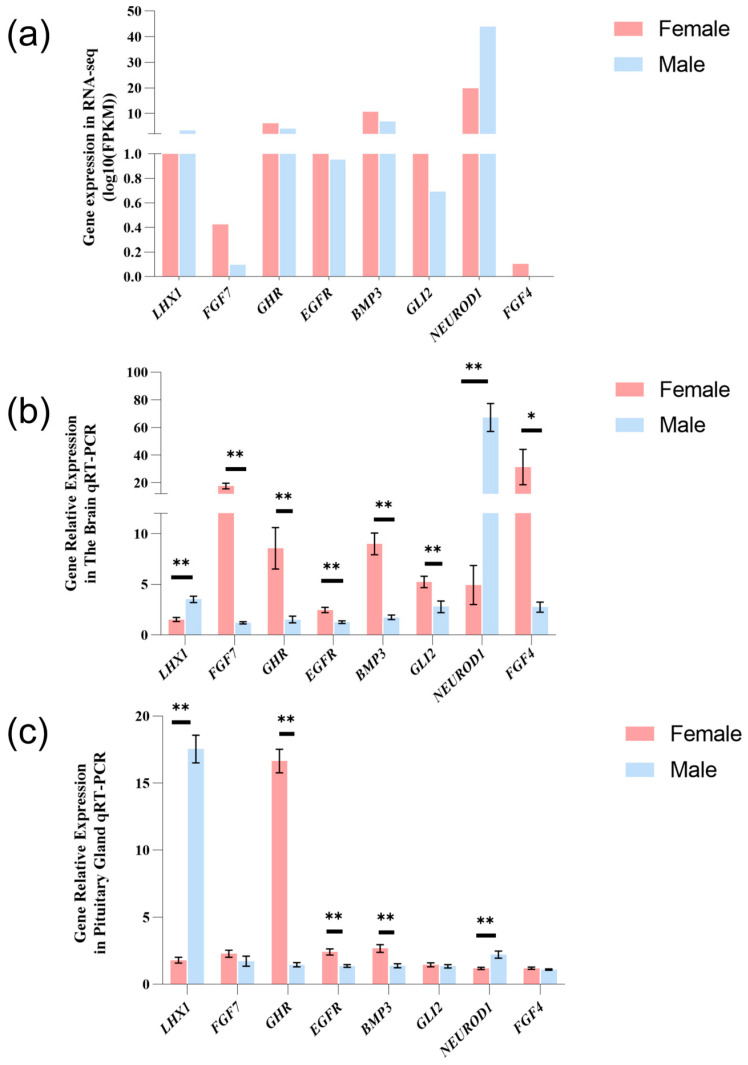
Gene expression analysis of eight selected genes in the brain and pituitary gland of adult male and female *P. sinensis* using RNA-seq and qRT-PCR. (**a**) RNA-seq analysis of gene expression levels (log10(FPKM)). qRT-PCR results showing the relative expression levels of these genes in (**b**) the brain and (**c**) the pituitary gland of adult male and female turtles. Significant differences are indicated by * (*p* < 0.05) and ** (*p* < 0.01). Red and blue bars represent female and male turtles, respectively.

In order to validate the accuracy of transcriptome sequencing, we randomly selected eight genes from DEGs for qPCR assay, including *LHX1*, *FGF7*, *GHR*, *FGF4*, *EGFR*, *BMP3*, *GLI2*, and *NEUROD1*. We used cDNA synthesized from the whole brain tissue pituitary glands of adult male and female *P. sinensis* as templates, with three biological replicates per tissue and three additional technical replicates per biological replicate. efα1 was used as the reference with the 2^−∆∆Ct^ method to calculate the relative expression level of candidate genes. Comparing the relative expression level of eight selected genes, most results of qPCR were consistent with the results of RNA-Seq (Figure 7).

## 4. Discussion

*P. sinensis* exhibits sexual dimorphism, which is reflected in a number of growth indicators, including dorsal carapace length, dorsal carapace width, and dorsal carapace height, as well as the obvious difference in secondary sexual characteristics between males and females, with the male tail exceeding the skirt and the female tail being inside the skirt [28]. Much literature supports the theory that dorsal armor is an important selection trait, and that sexually dimorphic differences in the dorsal armor of *P. sinensis* are important indicators for unisexual breeding considerations [29]. Tail length in *P. sinensis* is often used as a secondary sex character to determine the sex of *P. sinensis* in production. In addition, the brain is often used as an important tissue to screen for sexually dimorphic growth. Therefore, in this study, we analyzed the whole transcriptome of female and male brains of *P. sinensis*. The quality results of each library and the validation results of qRT-PCR indicate that we have established a high-quality and reliable transcriptome database. This provides a reliable database for our subsequent analyses. Subsequently, we screened 908 differential genes by differential basic analysis. Among these differential genes, we identified a number of genes related to growth, including *GHR* and *VEGFA*. First, we identified the growth-related gene *GHR*. The *GHR* gene is a receptor for growth hormone (GH), a central hormone in the biological axis that controls growth and development and is specifically responsible for growth promotion. In order to fulfill its physiological functions, growth hormone first binds to growth hormone receptors (GHRs) on the membrane surface of target cells, which mediate the entry of extracellular signals into the cell to produce a series of physiological effects that promote growth and development [30]. It has been shown that *GH* promotes growth and development of rainbow trout (*Salmo gairdneri*), resulting in increases in growth rate, weight gain, and length [31]. Previous studies have also revealed that the *GHR* gene promotes growth and development in Reeves’ turtle (*Chinemys reevesi*), and *GHR* was found to be minimally expressed in the male brain and highly expressed in the female brain of Reeves’ turtle [32], which is consistent with the results of this experiment. In the case of *VEGFA*, some studies have shown that the *VEGFA* gene has a role in influencing the early forebrain development in mice and promoting the growth and development of early mouse embryos [33]. It has also been shown that the *VEGFA* gene may play a role in the growth of *P. sinensis* [34]. Our results indicate that there are differences in gene expression patterns in the male and female brains of adult *P. sinensis*, which is consistent with previous findings in zebrafish [35,36], which also showed that the male and female brains differ in neuronal structure, namely the size, shape, and connectivity of neurons, which may contribute to the different gene expression patterns in female and male brains [37]. At the same time, we performed GO and KEGG pathway analysis and found some pathways related to growth. The growth-related gene *GHR* is enriched in the growth hormone synthesis, secretion, and action pathway, which directly affects the growth and development of *P. sinensis* [32]. The MAPK (mitogen-activated protein kinase) pathway plays an important role in the regulation of various biological processes, including the growth and development of different organisms, and *P. sinensis* is no exception. Studies have shown that the MAPK signaling pathway is critical for processes such as cell proliferation, differentiation, and response to environmental stresses, which are directly related to growth mechanisms [38]. Given that this gene and pathway play a very important role in the growth of the above species, but show a sexually dimorphic expression pattern in the brain of *P. sinensis*, we hypothesized that candidate genes and pathways regulate dimorphic expression in *P. sinensis*.

In addition, to identify genes that participate in both sex and growth, we performed GESA analysis and identified two differentially expressed genes, *LHX1* and *FGF7*. We screened for the LIM homology box family gene *LHX1*, a differential gene. It was demonstrated in a previous study that the interactors and transcriptional targets of *LHX1* are key to elucidating the multiple sets of factors involved in head development and building the head gene regulatory network [39]. Meanwhile, *LHX1* was found to be uniquely present in the testicular transcriptome of the Chinese sturgeon (*Acipenser sinensis*) [40]. We also screened for the *FGF7* gene, which belongs to the fibroblast growth factors (FGFs), a family of structurally related peptides that regulate processes such as cell proliferation, differentiation, and damage repair [41]. Fibroblast growth factors (FGFs) were discovered as early as 1974 [42] and can be classified as paracrine, endocrine, or autocrine, depending on their mode of action [43]. In addition to the autocrine type, FGFs exert their biological effects by binding to specific receptors, FGFRs [44]. In mammals, the *FGF7* gene has been identified to promote the migration of keratinocytes [45,46], as well as being involved in muscle cell differentiation, juvenile muscle development, and repair of damaged muscles in vitro [47], demonstrating a role for this gene in growth and development. In addition to this, *FGF7* was shown to play a role in *S. schlegelii* gonadal development, and the gene was highly expressed in *S. schlegelii* ovaries [47]. Therefore, these two genes may regulate sex-related pathways by participating in sex and growth pathways in the brain. In summary, these two genes play important roles in growth and sex pathways, and are also key genes involved in both growth and sex in *P. sinensis*, so it is reasonable to speculate that these two genes may be the key factors influencing sexually dimorphic growth in *P. sinensis*.

Furthermore, growth factors can combine with cis-acting elements, thereby regulating gene expression and participating in growth, development, and sex differentiation of organisms, so we identified and characterized transcription factors and investigated transcription factors and related proteins with potential interactions with *LHX1* and *FGF7* through PPI interactions. *NEUROD1* is a transcription factor involved in neural and endocrine regulation and has been implicated in the regulation of the hypothalamic–pituitary–gonadal axis, affecting reproductive and growth processes. Its expression may be associated with the regulation of growth and sexual development in *P. sinensis* [48]. The protein *FGF4* is particularly important during early embryonic development, and studies have shown that it plays a key role in mesoderm formation and differentiation of axial structures (e.g., spine, muscle, and skin) in vertebrates. Particularly in the early embryos of mice and vertebrates, the FGF signaling pathway influences the differentiation of the mesoderm in the central and paraxial axes and controls muscle and bone development [49]. *FGF4* also regulates the proliferation and differentiation of stem cells through activation of the MAPK signaling pathway and may be involved in the early development of the gonads [50]. Therefore, these two genes may be involved in the processes of sex and growth in *P. sinensis* by interacting with the above genes.

## 5. Conclusions

In this study, we performed transcriptome analysis of the brains of female and male *P. sinensis*. We performed GO and KEGG annotation on the obtained sets of differentially expressed genes and transcription factors, and screened out some genes and pathways associated with growth. In addition, by GSEA analysis, we screened out two genes, *LHX1* and *FGF7*, which were associated with both growth and sex differentiation, and we also used protein interactions analysis to screen out some genes associated with growth or sex differentiation. Finally, we screened eight genes and verified the expression levels of these eight genes in the brains of *P. sinensis* by qRT-PCR, which supported the reliability and accuracy of our transcriptome analysis. These findings provide valuable fundamental data for the study of sexually dimorphic growth in *P. sinensis*.

## Figures and Tables

**Table 1:** Overview of transcriptome sequencing data for each sample.

Sample	Raw Data (bp)	Clean Data (bp)	Q20 (%)	Q30 (%)	GC (%)	Unique Mapped (%)	Multiple Mapped (%)	Total Mapped (%)
Brain_F1	5,718,517,200	5,664,650,419	97.15	92.10	45.45	87.62	2.24	89.86
Brain_F2	5,827,686,300	5,782,101,559	96.96	91.70	44.29	87.93	1.94	89.87
Brain_F3	5,663,859,000	5,609,583,016	97.09	92.03	45.31	87.01	2.21	89.21
Brain_M1	5,643,272,400	5,597,172,355	97.05	91.86	45.01	87.32	2.15	89.47
Brain_M2	6,467,321,100	6,405,607,451	96.88	91.65	45.18	86.89	2.18	89.07
Brain_M3	5,997,436,200	5,939,819,117	97.06	91.96	45.84	86.27	2.23	88.50

## Data Availability

The raw data analyzed in this study can be downloaded from the National Center for Biotechnology Information (NCBI) databases (PRJNA1046242).

## Supplementary Materials

The following supporting information can be downloaded at: https://www.mdpi.com/article/10.3390/ani14233426/s1.
