# Supplementary material for: Sex-Dimorphic Differential Expression Profiles in the Brain of the Adult Chinese Soft-Shelled Turtle, Pelodiscus sinensis
Source: Animals (Basel). 2024 Nov 27;14(23):3426. doi: 10.3390/ani14233426 (PMC11640296; doi:10.3390/ani14233426)
Supplement: Supplementary file 1 [file animals-14-03426-s001.zip › Table S1 .pdf]

Table S1. All primers used in qRT-PCR analysis.

Type	Name	Sequences (5′-3′)
Gene	*LHX1*	F: CCCAAGACCCTTCCCAAGAC<br>R: CCTGTATGACGCGCATGTTG
	FGF7	F:CAGGAACAAGATATCCTGACTGA<br>R: AGTGCAGTGTTCTGGGTGTT
	GHR	F: CTCCTGGGAGTGGTTATGGC<br>R: TGTGTGGCCGTCCCAAAATA
	EGFR	F: GAGGTGGTGCTTGGCAATCT<br>R: GTTCCCTGAGTCCACTGGTT
	BMP3	F: ATGCTACTTGGAGTTGCCCC<br>R: CAAGGCCATTTTCACGCCAA
	GLI2	F: TAGAAACTTTCCCTCGCCCC<br>R: GCCACACCACTTTCGGACTA
	NEUROD1	F: GCCCTATGAGAGCCATTCCC<br>R:GGACAGGAACTCTGCATCCC
	FGF4	F: AATCTTGCTCCTGGGACTGC<br>R:AATCCCGTTGATTCTGCCGT
Reference gene	*efα1*	F: ACTCGTCCAACTGACAAGCCTC<br>R: CACGGCGAACATCTTTCACAG

F, forward primer; R, reverse primer;
